# 55179 An assessment of understandability and actionability in breast cancer survivorship print materials

**DOI:** 10.1017/cts.2021.731

**Published:** 2021-03-30

**Authors:** Pearman D. Parker, Arpan V. Prabhu, L. Joseph Su, Kristin K. Zorn, Carolyn Greene, Kristie B. Hadden, Jean C. McSweeney

**Affiliations:** 1 University of Arkansas for Medical Sciences; 2 College of Nursing; 3 College of Public Health; 4 College of Medicine

## Abstract

ABSTRACT IMPACT: Our results reveal a limited amount of breast cancer survivorship print materials as both understandable and actionable, and indicate a need to supplement material with personalized teaching. OBJECTIVES/GOALS: Using educational print material for young women breast cancer survivors (YBCS) is considered a best practice in patient teaching. Little is known about how well YBCS understand or act upon the material. The purpose of this study was to assess the understandability and actionability of commonly distributed breast cancer survivorship print materials. METHODS/STUDY POPULATION: We used an environmental scan approach to obtain breast cancer survivorship print materials available in eight outpatient oncology clinics and one electronic medical record used in a Midwestern state. Print materials were included if they were freely available to patients, were specific to breast cancer, provided detailed information about survivorship, and were directly given to patients by physicians or nurses. Print materials were excluded if topics were related to treatment, diagnosis, or prevention. All brochures, drug advertisements, and advertisements for support services were excluded. The understandability and actionability analyses of the breast cancer survivorship print materials were analyzed using Patient Education Materials Assessment Tool for Printable Materials (PEMAT-P). RESULTS/ANTICIPATED RESULTS: The environmental scan resulted in 82 individual print materials. After applying the inclusion and exclusion criteria, eight breast cancer survivorship print materials were included in the final sample. The final sample included two books, two patient education handouts from the electronic medical record, two multi-page booklets, and two pamphlets. The overall mean understandability score of the print materials was 68.9% ? 11.3 with a range of 47% to 80%. Five materials scored above the recommended 70% in understandability. The overall mean actionability score of the print materials was much higher at 81.3% ? 21.6 with a range of 67% to 100%. Five materials scored above 70% in actionability. However, only three of the eight materials scored above the recommended 70% in both understandability and actionability. DISCUSSION/SIGNIFICANCE OF FINDINGS: Limited breast cancer survivorship print materials exist as both understandable and actionable. Personalized instruction provided by oncology team members may be indicated to supplement the material. This additional teaching may help ensure survivors comprehend messages and act upon specific tasks as indicated in survivorship print material.

